# A Pilot Exploratory Study to Form Subgroups Using Cluster Analysis of Family Needs Survey Scores for Providing Tailored Support to Parents Caring for a Population-Based Sample of 5-Year-Old Children with Developmental Concerns

**DOI:** 10.3390/ijerph19020744

**Published:** 2022-01-10

**Authors:** Motohide Miyahara

**Affiliations:** 1 Department of Clinical Psychological Science, School of Medicine, Hirosaki University, Aomori 036-8564, Japan; motohide.miyahara@gmail.com; 2 Institute of Ars Vivendi, Ritsumeikan University, Kyoto 603-8577, Japan; 3 The Japan Centre for Evidence Based Practice, Osaka 565-0871, Japan

**Keywords:** needs assessment, cluster analysis, support groups, professional consultation, neurodevelopmental disorders

## Abstract

In a population-based developmental screening program, healthcare providers face a practical problem with respect to the formation of groups to efficiently address the needs of the parents whose children are screened positive. This small-scale pilot study explored the usefulness of cluster analysis to form type-specific support groups based on the Family Needs Survey (FNS) scores. All parents (N = 68), who accompanied their 5-year-old children to appointments for formal assessment and diagnostic interviews in the second phase of screening, completed the FNS as part of a developmental questionnaire package. The FNS scores of a full dataset (N = 55) without missing values were subjected to hierarchical and K-means cluster analyses. As the final solution, hierarchical clustering with a three-cluster solution was selected over K-means clustering because the hierarchical clustering solution produced three clusters that were similar in size and meaningful in each profile pattern: Cluster 1—high need for information and professional support (N = 20); Cluster 2—moderate need for information support (N = 16); Cluster 3—high need for information and moderate need for other support (N = 19). The range of cluster sizes was appropriate for managing and providing tailored services and support for each group. Thus, this pilot study demonstrated the utility of cluster analysis to classify parents into support groups, according to their needs.

## 1. Introduction

The symptomatic onset of neurodevelopmental disorders (e.g., attention-deficit/hyperactivity disorder, autism spectrum disorder, intellectual disabilities, specific learning disorder, and motor disorders) precedes school entry, alerting families, educators, and clinicians that the child may encounter problems in the behavioral, learning, and social domains at school [1]. To detect the early symptoms, developmental health screening has been recommended for identification, diagnosis, and prescription of individual and social interventions [2]. In Japan, since the enactment of the Act on Support for Persons with Developmental Disabilities in 2005, there has been increasing public awareness of support needs for 5-year-old children with neurodevelopmental disorders and their families before school entry at 6 years of age [3]. Public and private service providers in the medical, educational, and social welfare sectors have been trying to meet the needs of such children and their families [4]. However, individual needs vary widely at home, school, and in the community [5]; thus, a one-size-fits-all approach does not work to sufficiently meet all needs [6]. To effectively meet these diverse needs, needs-based support and identification of needs typologies would be useful [7]; how do we find the needs patterns?

In an ideal world, a local team of knowledgeable and experienced professionals would assess individual needs and preferences, then plan and provide joined-up services and support [8]. However, in reality, there is limited funding and resources; such ideal scrutiny of individual needs is not always possible [9] or cost effective [8]. A more realistic and practical option involves conducting a group survey, identifying the subtypes of needs typologies within the group, and providing tailored services and support [7,10]. As a “rule of thumb”, the number of community health support group members should range from 5 to 15 to ensure close attention and to enable a group to continue with some absentees [11]. A survey of such groups in the UK showed that the mean number of active attendees was approximately 15 [12]. Parents of children with severe or profound intellectual and developmental disabilities in the USA reported their experiences of attending meetings of group sizes varying from fewer than 10 to more than 20 members; they commonly preferred a group of 10 or fewer members [13]. Based on these studies, the present study attempted to form groups of similar sizes, with the number in each group ranging from 5 to 20.

The parent survey used in the present study is introduced in the following paragraphs, followed by a methodological discussion of the statistical identification of needs typology subtypes. Although the practical implementation of the survey and statistical procedures will be described under the heading of Materials and Methods, a methodological review within this introduction is essential to demonstrate the development of the survey and the absence of other similar tools; it is also essential to inform the reader of past studies that used cluster analysis for pediatric conditions. Thus, the survey material and the primary statistical method to be employed in this study are explained before introducing their practical details.

In 1988, Donald B. Bailey Jr. and Rune J. Simeonsson developed the Family Needs Survey (FNS) [14] to assist the subjective clinical judgment of interventionists with an easy-to-measure survey that requires no specialized training for families who have infants with disabilities. The FNS consists of 35 item statements, most of which include statements such as “I need more…” or “Our family needs help in…”.

Additionally, an open-ended question to specify the “five greatest needs as a family” who has an infant with a disability in cognitive and motor development was included. The 35 items were initially divided into six sections of different needs types, including: (1) information, (2) support, (3) explaining to others, (4) community services, (5) financial needs, and 6) family functioning. Each item was rated on a 5-point scale from “1 = Strongly Disagree” to “5 = Strongly Agree.” Although the survey developers found that an open-ended question was useful to gain additional information from 34 two-parent families, they concluded that the 35 items were sufficient to measure family needs.

In 1990, the FNS was revised and reorganized into 7 sections: (1) information, (2) family and social support, (3) financial needs, (4) explaining to others, (5) child care, (6) professional support, and (7) community services. The revised version was administered to 229 parents in 10 different states; analysis of the survey results confirmed that the survey was helpful for parents to communicate their needs to professionals and for professionals to understand the parents’ needs [15]. The FNS was equally accepted by both mothers and fathers, although 60% of the fathers and 40% of the mothers preferred the written survey approach to the interpersonal approach. In addition, the FNS was acceptable for minority and low-income parents. In the same year, the response format of the FNS was modified from a 5-point scale to a 3-point scale (1 = No; 2 = Not Sure; 3 = Yes) for the question, “Would you like to discuss this topic with a staff person from our program?”

For a quarter of a century since the development of the FNS in 1988, no researcher has substantially revised the FNS or developed a new survey to examine the needs of families who have children with disabilities. In 2013, Ueda and her group in Japan translated the FNS (1990 version) into Japanese and administered it to 1171 parents (719 mothers and 452 fathers) of children with disabilities ranging in age from 0 to 15 years [16]. These children were enrolled in institutions or special schools, suggesting that they had severe disabilities. The Japanese version of the FNS was examined for content validity by 130 healthcare professionals who worked with children with disabilities. The Japanese version was useful for parents of young children, consistent with the original FNS; it was also useful for parents of school-aged children. The content of the Japanese version of the FNS was considered reasonable by various healthcare professionals. The Japanese study also revealed differences between mothers and fathers of the same children, in that mothers expressed more of a need than did fathers for information, support, and explaining to others; however, they did not express greater financial needs. In addition, mothers of low and middle socioeconomic status indicated more needs than did mothers of high socioeconomic status. Thus, the Japanese version of the FNS demonstrated its usefulness in Japan for parents whose children had severe disabilities.

The utility of the FNS has also been demonstrated by parents who have children with cerebral palsy [10,17,18]. Of the three studies conducted by the same research group in the USA, one study [10] used cluster analysis and identified four profiles based on family needs: Cluster 1 (*n* = 294), low needs; Cluster 2 (*n* = 108), needs for child health; Cluster 3 (*n* = 114), needs for community and financial resources; Cluster 4 (*n* = 63), high needs. While four distinct cluster profiles were identified to “address the needs expressed by families” [10] (pp. 799), the number of cluster members was excessive for the establishment of interactive personalized support groups.

Concerning the family needs of neurodevelopmental disorders, only one study in Canada [7] has administered the FNS to the parents of children with autism spectrum disorder and reported FNS descriptive statistics. That study also examined the correlations between the demographic profiles and parental needs, but parental needs profiles were not identified based on the FNS.

To the best of our knowledge, no study has employed cluster analysis to identify family needs patterns in children with probable neurodevelopmental disorders to provide pattern-specific group services and support in the future. Therefore, the present exploratory study aimed to perform cluster analysis on the FNS to identify family needs typologies perceived by parents whose 5-year-old children participated in a population-based health screening for neurodevelopmental disorders and formed groups consisting of 5–20 parents.

## 2. Materials and Methods

This small, population-based, cross-sectional pilot study consisted of the parents of a cohort of 5-year-old children who attended the second phase of a screening program within Hirosaki City, Aomori Prefecture in Japan in 2020.

### 2.1. Participants and Procedures

Participants comprised 68 parents whose children attended the second phase of the Hirosaki Five-Year-Old Developmental Check-up (HFC) Study (See Mikami et al., 2020 [19] and Saito et al., 2020 [20] for details) in 2020. The first phase of the annual city-wide developmental screening consisted of a postal questionnaire survey concerning behavioral, emotional, motor, and social development. In the second phase, children with positive screening results underwent formal assessments and diagnostic interviews by healthcare professionals. During the second phase, a Japanese version of the FNS (more details in the next section below) was included in the survey package with other assessment instruments, such as questionnaires for attention-deficit/hyperactivity disorder and autism spectrum disorder. Ethical approval was obtained from the Committee of Medical Ethics of the Hirosaki University Graduate School of Medicine (2018-168-1).

### 2.2. Measures

The Family Needs Survey (FNS, 1990 version) was translated into Japanese and used as a tool to collect data for this study. When we planned our translation, we were unaware of the existence of the Japanese version of the FNS that had already been translated by Ueda et al., (2013) [16], based in Osaka, Japan. For the present study, a native Japanese researcher, who has worked as a psychologist in English-speaking countries for more than 30 years, translated the FNS into standard Japanese. The draft translation was checked and slightly modified for ease of legibility and appropriateness in the local context by a psychiatrist in charge of the HFC study. The modified translation was further examined for cultural adaptation by a psychologist on the HFC study team who had been born and raised in a local province. We decided not to pursue back translation because our quality evaluation process was considered more effective than back translation [21]. Only when we requested permission from the authors of the FNS to translate it into Japanese and use it for our study, did we learn that the Japanese translation had existed since 2013. The translators in Osaka shared their version, which we compared with our version. Two items that the translators changed, from “a church or synagogue” into “religious services” and from “a minister, priest, or rabbi” into “religious workers,” were modified similarly in our version: “short-term childcare when necessary” and “individuals from religious organizations”. Although there were several minor differences in phrasing, we retained our translation for the suitability of the local socio-cultural context.

To the original two open-ended questions, “Please list other topics or provide any other information that you feel would be helpful to discuss” and “Is there a particular person with whom you would prefer to meet?”, we added “Please comment on this survey”, in accordance with the method used by Bailey and Blasco (1990) [15] to examine parents’ perspectives concerning the FNS. Our Japanese translation is shown in Appendix A.

### 2.3. Data Analysis

SPSS Statistics for Windows, version 27 software (IBM Corp., Armonk, NY, USA) was used for the statistical analysis. First, missing data were excluded, and effective percentages of the three responses to each of the 35 FNS items were computed to present the primary quantitative descriptive data. Second, the verbatim presentation of answers to the three open-ended questions was summarized in a table. Third, Ward’s hierarchical agglomerative method and the K-means iterative partitioning method were used to compare and determine reasonably similar numbers of cluster memberships for the formation of potential support groups. The hierarchical cluster analysis began with each parent as a cluster and was successively linked to other parents or clusters until all parents were contained in a single cluster; Ward’s minimum variance method was selected to form similar numbers of cluster members [22] to plan efficient needs-type-specific support group sessions. The K-means iterative partitioning method [22] was used to reassign the parents, beginning with two clusters, until reasonably similar numbers of cluster memberships were discovered for potential group support sessions. Finally, the cluster profiles and multidimensional scaling (MDS) of the final solution were graphically examined to interpret the scoring patterns and visualize the distances between the clusters and cluster members [23].

## 3. Results

### 3.1. Descriptive Analysis of the FNS

Table 1 shows the effective percentages, consisting of the percentages of three responses (no need for help, uncertainty, or a definite need) for each FNS item; the number of parents with missing data is subtracted from the denominator. Among the seven FNS sections, missing responses were less frequent (<3) in the first three sections than in the last four sections (>3). The section that reflected the greatest needs was the information section: more than 50% of parents expressed a definite need for help with all items in the section. This was followed by the community services section: more than 45% of parents expressed a definite need for help with all items in the section. In the professional support section, the need for “more time to talk to health professionals” was high: 45% of parents expressed a definite need for help. In contrast, a complete absence of definite need was indicated for “meeting with individuals from religious organizations.” Low definite need (<5% of parents) was also observed for “family recreational activities and basic expenses.” Overall, complete datasets from 55 of the 68 parents were subjected to cluster analyses.

### 3.2. Verbatim Presentation of Open-Ended Questions

Table 2 shows the answers, provided by a total of 18 parents, to the three open-ended questions. The first question, which asked respondents to list topics that are not covered in the 35 forced-choice questions, enabled parents to freely mention their concerns. All five answers contextualized specific concerns pertaining to each of their children and the needs of the parents. In response to the second question concerning specific personnel to consult with, two parents requested the same doctor and one parent requested a public health nurse. In response to the third question, there was a split between comments that were positive and comments that indicated the potential for improvement; some parents found that the questions were easy to understand and helped their preparation for clinical interviews, while others felt unprepared to answer and felt that the forced-choice questions were difficult to rate.

### 3.3. Determination of the Numbers of Clusters and Members Per Cluster

**Figure 1:**
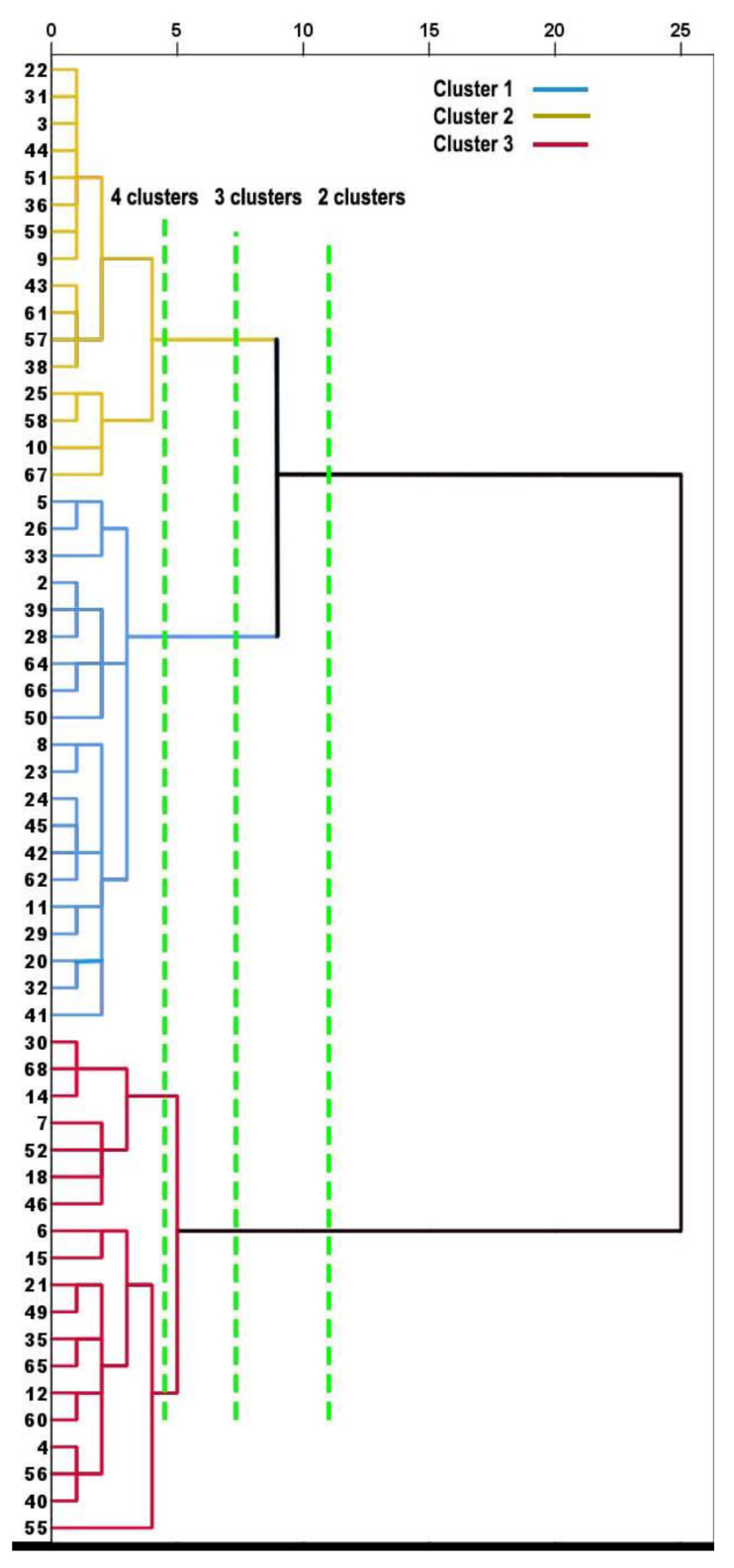
Dendrogram depicting hierarchical agglomerative clustering with Ward’s linkage into 2, 3, or 4 cluster solutions. The three-cluster solution is color coded.

The numbers of clusters and members per cluster were explored by performing cluster analyses of the hierarchical method and the K-means method. The hierarchical agglomerative method with Ward’s linkage produced the dendrogram shown in Figure 1, which suggested that a three-cluster solution would distribute similar numbers of cluster members. The hierarchical and K-means cluster analyses for 2, 3, and 4 cluster solutions (Table 3) indicated that the three-cluster solution yielded from the hierarchical agglomerative clustering was an optimal final solution for grouping similar numbers of cluster members.

### 3.4. Needs Profiles and Distances between the Profiles

**Figure 2:**
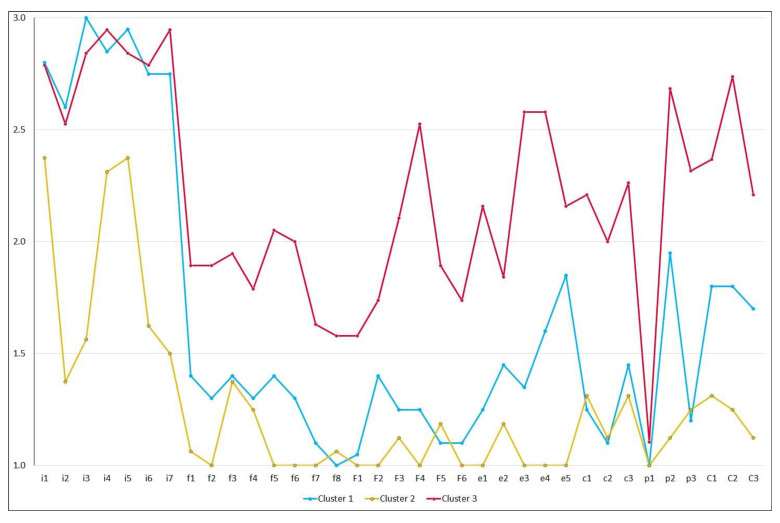
Three unique needs profiles of the final three-cluster solution based on the mean scores for each need item in the Family Needs Survey.

**Figure 3:**
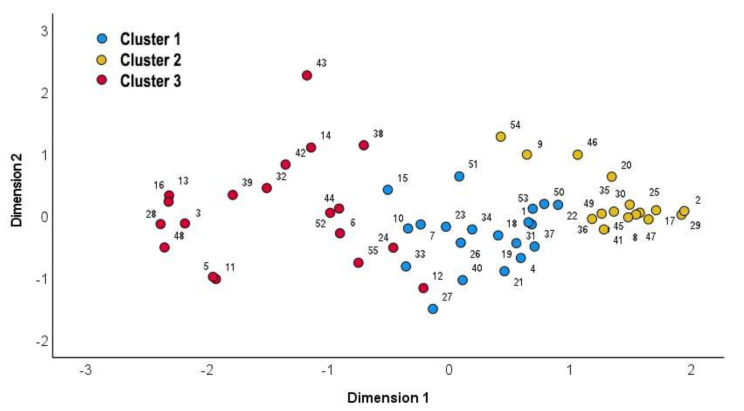
Multidimensional scaling (MDS) of the final three-cluster solution.

Based on the mean scores for each need item, three unique needs profiles are depicted in Figure 2. Parents in Cluster 1 (*n* = 20) had a high need for information support and a moderate need for expert reading materials and professional consultation sessions. Parents in Cluster 2 (*n* = 16) had a moderate need for information support and a low need for other areas of support. Parents in Cluster 3 (*n* = 19) had a high need for information support and a moderate need for other areas of support. Differences among clusters were significant (*p* < 0.001) in all items, except the item which asked about the need for “meeting with individuals from religious organizations.” The result of multidimensional scaling (MDS) of the final three-cluster solution in Figure 3 distinguished between members in Clusters 1 and 2 and between members in Clusters 2 and 3; however, it highlighted the proximity between members in Cluster 1 (Parents 15, 27, and 33) and Cluster 3 (Parents 12 and 24) located from −1 to 0 on Dimension 1.

## 4. Discussion

This pilot exploratory study applied cluster analysis to the Family Needs Survey (FNS, 1990 version) scores obtained from 68 parents to form support groups with sizes ranging from 5 to 20 members through the identification of parental needs subtypes concerning their 5-year-old children with probable neurodevelopmental disorders. Hierarchical agglomerative clustering with Ward’s linkage method classified 55 of 68 parents into three groups, which consisted of similar numbers of parents who expressed distinct needs patterns.

The importance of forming support groups, as a corollary of the importance of the present study, was confirmed by the observation that all three clusters displayed profiles of high to moderate needs for information. This finding concerning the need for information is consistent with the results of two previous studies that administered the FNS to the parents of children with autism spectrum disorder [7] and the parents of children with cerebral palsy [10]. In the study concerning autism spectrum disorder, 94% of the parents needed information; the most frequently identified needs were information related to services available now (82% of participants) and the future (79% of participants). In the study concerning cerebral palsy, four clusters were identified based on the FNS, and two of the four clusters indicated high information needs. Specifically, Cluster 2: Needs for child health included 18% of parents with high needs for information that explains the child’s condition to others, while Cluster 4: High needs included 11% of parents with very high needs in all areas of needs, including the need for information.

The unmet need for information is striking, considering the contemporary digitally connected era, when the effects and usefulness of online informational and social support have been extensively reported [24,25], with particular demand in the context of the current coronavirus disease 2019 (COVID-19) pandemic [26,27]. The answers to the open-ended questions of the FNS in Table 2 provide clues concerning the type of information sought by the parents. Specific contextualized concerns pertaining to each of their children were mentioned, which the forced-choice format could not sufficiently explore. Dr. X was nominated by two respondents. Collaborators at the site of the health check-up study discussed possible reasons for this and concluded that it was extremely easy for parents to speak to Dr. X in an uninhibited manner. Overall, the findings indicate that a need remains for an in-person consultation service, which online platforms may not be able to fully replace.

While there may be a need for in-person support, online consultations are necessary during the current COVID-19 pandemic [26,27]. Individual, real-time, interactive formats are preferred for online services and support; such a format may be employed, depending on the resources available [24]. If this format is unavailable, needs-type-specific group support sessions can be designed based on the needs patterns identified by the cluster analysis of the FNS. The proximity of some members in Cluster 1 and Cluster 3 in Figure 2 indicates that these group members are interchangeable. Thus, the statistical analysis approach in the present study will be useful for new FNS datasets in any local or global community that is planning to provide needs-type-specific support for the parents of children who have developmental concerns. Finally, the proportion of incomplete datasets was approximately 20%; the higher missing responses in the last four sections (>3) than in the first three sections (<3), coupled with the responses to the last open-ended questions by 18 of the 68 parents, were suggestive of a fatigue effect. Therefore, future research should explore alternative non-survey methods for the overlooked subsample to communicate their needs [16].

## 5. Conclusions

The present pilot study demonstrated the usefulness of hierarchical cluster analysis with Ward’s linkage for forming groups of appropriate sizes to provide needs-type-specific group services and support by identifying needs typologies of parents who have children with developmental concerns. The study findings are limited to parents involved in their children’s second phase of the population-based developmental check-up. When the diagnostic assessment results are available, parents should be invited for needs-type-specific consultation and support groups; the feasibility and usefulness of the group support approach should be investigated. Each subgroup of parents may have a set of common features concerning demographic and socioeconomic characteristics [10], the child’s diagnostic status, and the parent’s stress level [5]. It will be important for future research to identify such covariates to meet specific needs. Furthermore, parents who have difficulties responding to the survey should have opportunities for expressing their needs outside of the survey platform [16].

## Figures and Tables

**Table 1:** Effective percentages of responses and numbers of missing responses according to item for all parents (N = 68).

Section Abbreviated Item Description	Definitely Do Not Need Help	Not Sure	Definitely Need Help	Number of Missing Response
1. Information				
1) How children grow and develop	10.6	10.6	78.8	2
2) How to play or talk with my child	39.4	4.5	56.1	2
3) How to teach my child	22.7	3.0	74.2	2
4) Handling child’s behavior	13.2	4.4	82.4	0
5) Child’s condition or disability	11.9	10.4	77.6	1
6) Current services	25.0	11.8	63.2	0
7) Future services	20.9	16.4	62.7	1
2. Family and Social Support				
1) Someone in my family to talk to	65.7	19.4	14.9	1
2) More friends to talk to	71.6	16.4	11.9	1
3) More time for myself	59.7	25.4	14.9	1
4) Spouse	62.7	20.9	16.4	1
5) Discussing problems/reaching solutions	59.7	22.4	17.9	1
6) Supporting each other	66.7	16.7	16.7	2
7) Household and child care tasks	79.1	13.4	7.5	1
8) Recreational activities	80.6	14.9	4.5	1
3. Financial				
1) Basic expenses	80.6	14.9	4.5	1
2) Special equipment	69.7	18.2	12.1	2
3) Therapy, day care services	64.2	20.9	14.9	1
4) Job counseling	59.7	16.4	23.9	1
5) Babysitting/respite care	73.1	13.4	13.4	1
6) Toys	74.6	16.4	9.0	1
4. Explaining to Others				
1) My parents or my in-laws	68.3	15.9	15.9	5
2) Siblings	69.8	14.3	15.9	5
3) Friends/neighbors/strangers	57.8	17.2	25.0	4
4) Other children	52.4	23.8	23.8	5
5) Reading material about other families	52.4	22.2	25.4	5
5. Child Care				
1) Baby-sitter/respite care	64.5	11.3	24.2	6
2) Day care program or preschool	75.4	11.5	13.1	7
3) Short-term care	63.5	6.3	30.2	5
6. Professional Support				
1) Individuals from religious organizations	96.8	3.2	0.0	5
2) Health care professionals (psychologist/social worker/psychiatrist)	48.4	6.3	45.3	4
3) Time to talk to my child’s teacher or health care professional	59.4	23.4	17.2	4
7. Community Services				
1) Other parents who have a child like mine	28.6	25.4	46.0	5
2) Doctor	38.1	15.9	46.0	5
3) Dentist	28.1	10.9	60.9	4

**Table 2:** Answers to the open ended questions of the Family Needs Survey.

1. Please list other topics or provide any other information that you feel would be helpful to discuss.
My child repeat doing the things that are told not to do. My child hits. Forgetful and distractive.
My child is not good at thinking independently. Compared to other children of the same age, my child speaks less. Colors are sometimes ambiguous. Poor at drawing pictures. Sings well.
About how my child behaves at the kindergarten, home, and in my presence and in my absence.
About the gaze when speaking to others. Recent observation of exaggerated eye blinking and twisted mouth. Maybe related to my work issues.
Would it be possible to receive support for daily living and homecare?
2. Is there a particular person with whom you would prefer to meet?
Dr X (who conducted a clinical interview), the health nurse in charge
Dr X
3. Please comment on this survey.
There is no clear diagnosis yet, so I didn’t know what to talk or consult about.
I was surprised by some questions.
The words used in the survey were easy to understand.
There are some items that are difficult to answer with "yes", "no", "not sure".
My daughter used to be restless, but she is settled these days. So, there is no issue that I need to consult about.
I hope to have inexpensive easy access to information about my child and about how to reduce mental and physical loads.
At the moment, there is nothing I am willing to consult. So, I don’t understand very well.
There were questions that my answers don’t fall on the scale or partially fall on the scale. It took me time to answer those questions.
The survey helped me to organize what I wanted to talk about before I talked with the staff. So, I thought it was very good.

**Table 3:** The numbers of members per cluster for the 2, 3, and 4 cluster solutions produced by two cluster analysis methods.

Method of Cluster Analysis	Cluster 1	Cluster 2	Cluster 3	
Hierarchical agglomerative method with Ward’s linkage	20	16	19	
K-mean cluster analysis solution after 4 iterations	24	16	12	
2 cluster solutions				
Method of cluster analysis	Cluster 1	Cluster 2		
Hierarchical agglomerative method with Ward’s linkage	36	19		
K-mean cluster analysis solution after 4 iterations	23	32		
3 cluster solutions				
Method of cluster analysis	Cluster 1	Cluster 2	Cluster 3	
Hierarchical agglomerative method with Ward’s linkage	20	16	19	
K-mean cluster analysis solution after 4 iterations	24	16	12	
4 cluster solutions				
Method of cluster analysis	Cluster 1	Cluster 2	Cluster 3	Cluster 4
Hierarchical agglomerative method with Ward’s linkage	20	26	12	7
K-mean cluster analysis solution after 4 iterations	20	29	5	1

## Data Availability

The data are not publicly available because of privacy reasons.
